# *Neoehrlichia mikurensis* in Ticks and Tick-Bitten Persons, Sweden and Finland, 2008–2009

**DOI:** 10.3201/eid3111.241850

**Published:** 2025-11

**Authors:** Emilia Hero, Malin Lager, Pia Forsberg, Per-Eric Lindgren, Anna J. Henningsson, Peter Wilhelmsson

**Affiliations:** Linköping University, Linköping, Sweden (E. Hero, P. Forsberg, P.-E. Lindgren, A.J. Henningsson, P. Wilhelmsson); Region Jönköping County, Jönköping, Sweden (M. Lager, P.-E. Lindgren, A.J. Henningsson, P. Wilhelmsson)

**Keywords:** *Neoehrlichia mikurensis*, vector-borne infections, bacteria, prospective studies, emerging tick-borne diseases, tick-borne, neoehrlichiosis, immunocompetent, ticks, human blood samples, clinical manifestations, real-time PCR, Sweden, Finland

## Abstract

By using PCR testing, we found *Neoehrlichia mikurensis* DNA in 1.1% of ticks removed from persons in Sweden and Finland. Symptoms developed in 2 immunocompetent persons. Despite low transmission risk, infection can occur after short tick attachment. Our findings highlight the need to consider *N. mikurensis* in patients with unexplained symptoms after tick bite.

*Neoehrlichia mikurensis* is an emerging tickborne pathogen, mainly transmitted by *Ixodes ricinus* ticks. The human infection known as neoehrlichiosis was first reported in Sweden in 2010 and has been documented in several countries in Europe (*1*,*2*). Neoehrlichiosis affects both healthy and immunocompromised persons, and more severe symptoms, such as vascular complications, are typically seen in immunocompromised patients (*3*). Diagnosis is challenging because of vague symptoms, low awareness, and lack of commercial diagnostic tests. PCR is the primary method of detection, because *N. mikurensis* is difficult to culture and no serologic tests are available. The true prevalence of *N. mikurensis* in humans and ticks remains unclear, although its DNA has been detected in both throughout Europe (*4*,*5*).

We investigated the prevalence of *N. mikurensis* after tick bites in northern Europe, aiming to detect its DNA in ticks and human blood and to assess symptoms among participants. The samples were originally collected in 2008–2009, when *N. mikurensis* was not yet recognized as a human pathogen and appropriate diagnostic tools were lacking; therefore, this analysis could not be conducted until recently. Despite being >15 years old, the data offer valuable insight into *N. mikurensis* ecology and transmission. Although epidemiology might have shifted, the underlying biologic mechanisms of pathogen transmission likely remain unchanged, ensuring continued relevance.

## The Study

We analyzed data from the Tick-Borne Diseases (TBD) STING study (*6*,*7*), a prospective multicenter study conducted in Sweden and on the Åland Islands, Finland (Figure). During May 2008–November 2009, primary healthcare centers enrolled 1,425 healthy tick-bitten adults (>18 years of age; median age 63, range 19–92) through public advertisements. The study was approved in 2008 by the Regional Ethical Review Board in Linköping, Sweden, and the Åland Health Care Ethics Committee.

**Figure:**
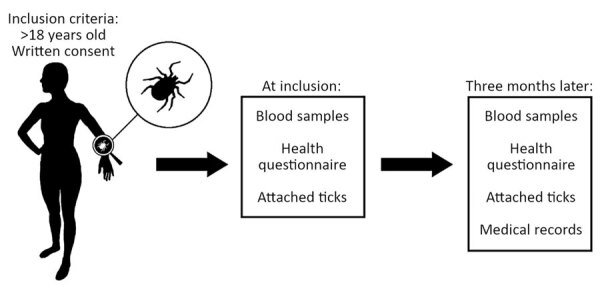
Schematic overview of the Tick-Borne Diseases STING study during 2008–2009 in Sweden and Finland (*6*), in which tick-bitten persons >18 years of age gave written informed consent to participate, submitted removed tick(s), provided a blood sample, and completed a questionnaire. At a follow-up visit 3 months later, they provided a second blood sample and completed a second questionnaire. Any additional tick(s) found attached during the study period were also submitted and available medical records were reviewed.

At study inclusion, participants submitted removed tick(s), provided a blood sample, and completed a questionnaire on tick exposure, prior tickborne diseases, current health status, and medication use (*8*). Participants on antimicrobial drugs or immunosuppressive therapy were excluded. A follow-up visit 3 months later included providing a second blood sample and filling out a questionnaire about symptoms experienced since inclusion and if the participant had sought medical care (*8*). Medical records were reviewed when relevant. Participants also submitted any additional ticks found attached during the 3-month study period.

For this study, we photographed each tick and identified it by species, developmental stage, and sex. We estimated feeding duration for nymphs and adult females (*9*). We homogenized ticks, extracted total nucleic acids, and reverse-transcribed to cDNA (*7*). Participant plasma was collected in EDTA tubes (*6*), and we extracted DNA from participants whose ticks were PCR-positive for *N. mikurensis*.

We screened tick cDNA for *N. mikurensis* by using a SYBR Green real-time PCR targeting the *16S* rRNA gene (*10*) and confirming positive results by using a TaqMan real-time PCR targeting the *groEL* gene (*11*). We analyzed plasma DNA from participants bitten by positive ticks by using the same TaqMan assay. We confirmed PCR products by sequencing (Macrogen Inc., https://www.macrogen.com) and verified sequences with BLAST (https://blast.ncbi.nlm.nih.gov). We used a previously confirmed *N. mikurensis*-positive cDNA sample as a positive PCR control (*10*).

Of the 1,644 *I. ricinus* ticks analyzed, 18 (1.1%) were positive for *N. mikurensis* in both PCRs (Table 1). No DNA was found in larvae. Prevalence in nymphs was 1.1% and in adult females was 1.3%. The ticks were collected from participants in various regions in Sweden and on the Åland Islands (Appendix Table). Of the 18 participants bitten by PCR-positive ticks, 2 (11%) tested positive for *N. mikurensis* (Table 2).

**Table 1:** The prevalence of *Neoehrlichia mikurensis* determined in 1,644 *Ixodes ricinus* ticks collected from 1,425 humans who were bitten in Sweden or on the Åland Islands, Finland, during May 2008–November 2009

Developmental stage of the tick	Total no. ticks analyzed	No. (%) *N. mikurensis* PCR-positive ticks*
Adult females	392	5 (1.3)
Adult males	7	0
Nymphs	1,138	13 (1.1)
Larvae	69	0
Undetermined†	38	0
Total	1,644	18 (1.1)

*PCR-positive tick was defined as a tick that tested positive for *N. mikurensis* in both the SYBR Green and TaqMan real-time PCR assays. †Tick developmental stage remains undetermined because of damages as previously described (*7*).

**Table 2:** Characteristics of participants with *Neoehrlicha mikurensis* DNA detected in their plasma in a study of ticks and tick-bitten persons, Sweden and Finland, 2008–2009.

Participant sex	Participant age, y	Tick developmental stage	Tick feeding time, h‡	PCR results§	Self-reported symptoms¶	Sought medical care
Female*	41	Nymph	<24	Positive/negative	Headache, fatigue, radiating pain, myalgia, numbness	Yes
Female†	68	Nymph	43	Negative/positive	Fatigue, neck pain, myalgia, numbness	No

*Participant was recruited to a primary healthcare center in southernmost Sweden (Appendix Figure). †Participant was recruited to a primary healthcare center in south-central Sweden (Appendix Figure). ‡Estimation of the duration of blood feeding was calculated as described previously (*9*). §Plasma samples collected during initial inclusion and then at follow-up visit. ¶Self-reported symptoms described in the questionnaire that was completed during the follow-up meeting 3 months after the tick bite.

The first positive participant was a 41-year-old otherwise healthy woman from southern Sweden who tested positive at inclusion. She had been bitten by a *N. mikurensis*–positive nymph that had fed for <24 hours. She reported symptoms including headache, fatigue, numbness, radiating pain, and myalgia. Eleven days later, she was bitten by another tick, which was not submitted for analysis. It was unclear whether her symptoms were related to the first or second tick bite. During the study period, she sought medical care for and was diagnosed with myalgia and calf muscle cramps, for which she was treated with quinine tablets (100 mg) administered as needed.

The second positive participant was a 68-year-old otherwise healthy woman from south-central Sweden, who tested positive at the 3-month follow-up. She had been bitten by a *N. mikurensis*–positive nymph that had fed for ≈40 hours. She reported fatigue, neck pain, myalgia, and numbness but did not seek medical care. She was bitten by 3 additional ticks during the study period, of which only 1 was submitted to the TBD STING study; it tested negative for *N. mikurensis*.

Sequencing confirmed *N. mikurensis* DNA in both the ticks and the blood samples from the 2 participants. The remaining 16 participants bitten by PCR-positive ticks tested PCR-negative for *N. mikurensis* in both the inclusion and follow-up blood samples. Out of those participants, 9 reported no symptoms during the study period and 2 did not respond to the follow-up questionnaire. The remaining 5 reported nonspecific symptoms, including muscle and joint pain (n = 5), headache (n = 2), fatigue (n = 2), neck pain (n = 1), vertigo (n = 2), and numbness (n = 2).

## Conclusions

We detected *N. mikurensis* DNA in 1.1% of *I. ricinus* ticks that had bitten humans in Sweden and Finland, indicating a low prevalence in the study areas compared with those reported in other regions of Sweden (*10*,*12*). Two participants bitten by PCR-positive ticks also tested positive for *N. mikurensis* in blood samples and reported symptoms consistent with neoehrlichiosis, despite being immunocompetent. Those findings suggest that, although the overall risk for infection after a tick bite is low, transmission can still occur even after short tick attachment times (<24 h) and in persons without known immunosuppression.

The prospective design of the TBD STING study, large number of samples, and standardized follow-up strengthen the reliability of our findings. The use of a 2-tier PCR targeting the *16S* rRNA and *groEL* genes and then sequencing ensured high specificity of the detection assay. By linking infected ticks to the person they had bitten, the study provided an opportunity to assess the risk for transmission under natural conditions.

Although PCR detects bacterial DNA rather than viable organism, its sensitivity depends on having enough *N. mikurensis* DNA present in the blood sample. Because *N. mikurensis* likely causes low-level bacteremia because of its tissue tropism (*13*,*14*), the pathogen might not always be detectable in blood samples, leading to false-negative results and potential underdiagnosis. Therefore, the symptoms reported by PCR-negative participants bitten by positive ticks might be related to *N. mikurensis* exposure. Furthermore, PCR cannot detect seroconversion or immune responses, and the absence of serologic tools limits the ability of this study to identify resolved infections.

The absence of *N. mikurensis* DNA in tick larvae supports evidence that transovarial transmission is unlikely (*10*,*12*,*15*), whereas similar prevalence in nymphs and adult females suggests stable infection rates across stages, informing our understanding of pathogen maintenance in tick populations. The relevance of our results likely extends beyond the specific regions studied. Therefore, in areas where *N. mikurensis* has been detected in ticks, clinicians should consider this pathogen in the differential diagnosis of patients with unexplained symptoms after a tick bite.

Appendix Additional information about *Neoehrlichia mikurensis* in ticks and tick-bitten persons, Sweden and Finland, 2008–2009.
