# Supplementary material for: Neoehrlichia mikurensis in Ticks and Tick-Bitten Persons, Sweden and Finland, 2008–2009
Source: Emerg Infect Dis. 2025 Nov;31(11):2149–52. doi: 10.3201/eid3111.241850 (PMC12704533; doi:10.3201/eid3111.241850)
Supplement: Appendix — Additional information about Neoehrlichia mikurensis in ticks and tick-bitten persons, Sweden and Finland, 2008–2009. [file 24-1850-Techapp-s1.pdf]

Article DOI: https://doi.org/10.3201/eid3111.241850

EID cannot ensure accessibility for supplementary materials supplied by authors. Readers who have difficulty accessing supplementary content should contact the authors for assistance.

Neoehrlichia mikurensis in Ticks and Tick-Bitten Persons, Sweden and Finland, 2008–2009

Appendix

Appendix Table. The prevalence of *Neoehrlichia mikurensis* determined in 1,644 *Ixodes ricinus* ticks collected from 1,425 humans who were bitten in different regions of Sweden and on the Åland Islands, in 2008 and 2009 respectively.

	No. of PCR-positive ticks out of total no. of ticks examined (%)		
Geographical region	2008	2009	Both years
Northern Sweden	0 of 4 (0.0)	0 of 11 (0.0)	0 of 15 (0.0)
South-Central Sweden	2 of 404 (0.5)	4 of 277 (1.4)	6 of 681 (0.9)
Southernmost Sweden	1 of 125 (0.8)	8 of 276 (2.9)	9 of 401 (2.2)
Åland Islands	2 of 329 (0.6)	1 of 218 (0.5)	3 of 547 (0.5)
Total	5 of 862 (0.6)	13 of 782 (1.7)	18 of 1644 (1.1)

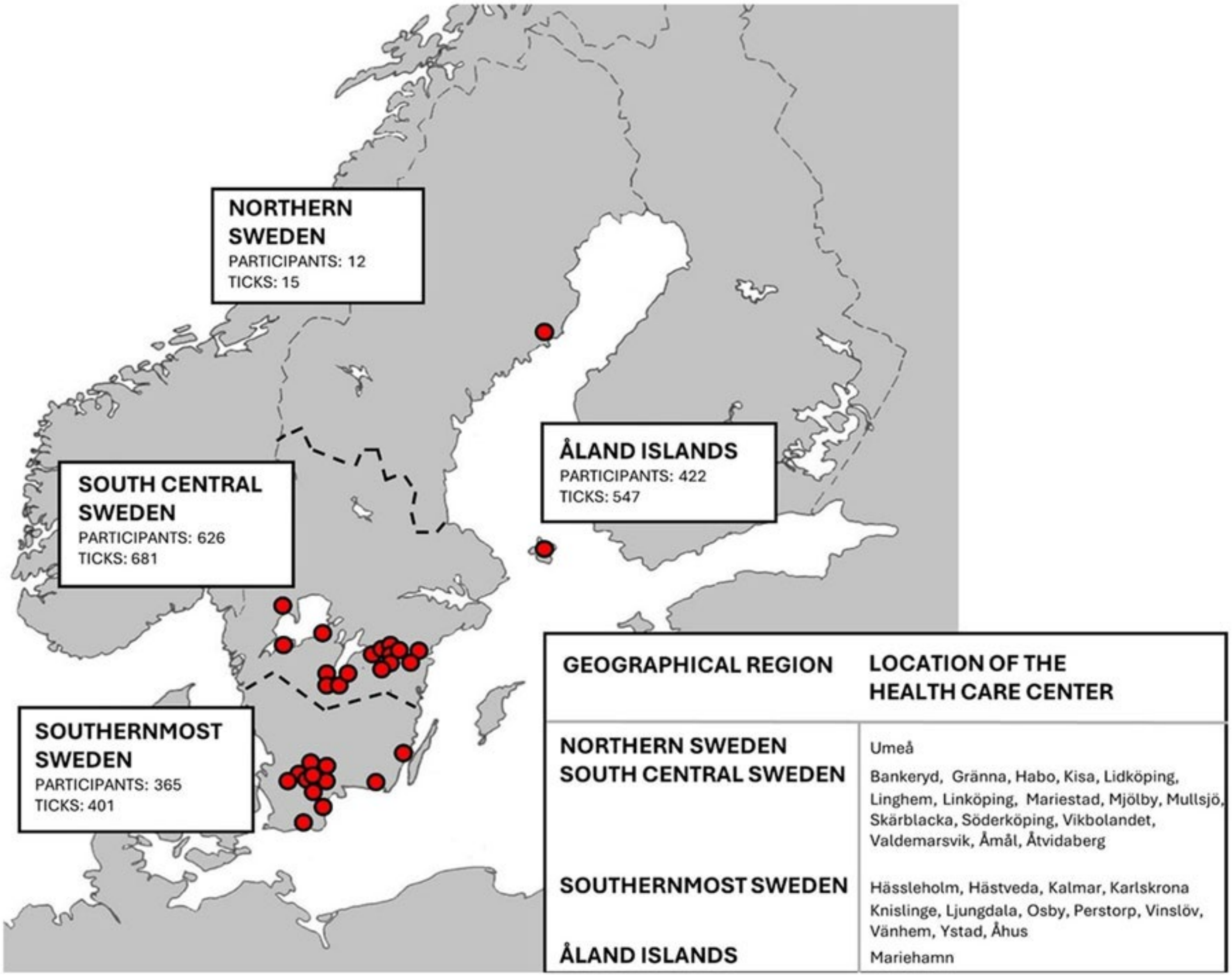

Appendix Figure. Number of participants included in this study and the ticks they collected during 2008 and 2009 are depicted across various geographical regions of Sweden (Northern, South-Central, and Southernmost Sweden) including the Åland Islands. The figure further delineates the locations of the recruited healthcare facilities within these regions.
